# Impact of a Shorter Brine Soaking Time on Nutrient Bioaccessibility and Peptide Formation in 30-Months-Ripened Parmigiano Reggiano Cheese

**DOI:** 10.3390/molecules27030664

**Published:** 2022-01-20

**Authors:** Mattia Di Nunzio, Cecilia Loffi, Elena Chiarello, Luca Dellafiora, Gianfranco Picone, Giorgia Antonelli, Clarissa Di Gregorio, Francesco Capozzi, Tullia Tedeschi, Gianni Galaverna, Alessandra Bordoni

**Affiliations:** 1 Department of Food, Environmental and Nutritional Sciences (Defens), University of Milan, Via Celoria 2, 20133 Milan, Italy; 2 Department of Food and Drugs, University of Parma, Parco Area delle Scienze 95/A, 43124 Parma, Italy; cecilia.loffi@unipr.it (C.L.); luca.dellafiora@unipr.it (L.D.); tullia.tedeschi@unipr.it (T.T.); gianni.galaverna@unipr.it (G.G.); 3 Department of Agricultural and Food Sciences (DISTAL), University of Bologna, Piazza Goidanich 60, 47521 Cesena, Italy; elena.chiarello2@unibo.it (E.C.); gianfranco.picone@unibo.it (G.P.); giorgia.antonelli4@unibo.it (G.A.); francesco.capozzi@unibo.it (F.C.); alessandra.bordoni@unibo.it (A.B.); 4 Interdepartmental Centre for Industrial Agri-Food Research (CIRI), University of Bologna, Piazza Goidanich 60, 47521 Cesena, Italy; clarissadigregorio@gmail.com

**Keywords:** Parmigiano Reggiano cheese, in vitro digestion, bioaccessibility, bioactive peptides

## Abstract

Reducing the salt content in food is an important nutritional strategy for decreasing the risk of diet-related diseases. This strategy is particularly effective when applied to highly appreciated food having good nutritional characteristics, if it does not impact either upon sensory or nutritional properties of the final product. This work aimed at evaluating if the reduction of salt content by decreasing the brine soaking time modifies fatty acid and protein bioaccessibility and bioactive peptide formation in a 30-month-ripened Parmigiano Reggiano cheese (PRC). Hence, conventional and hyposodic PRC underwent in vitro static gastrointestinal digestion, and fatty acid and protein bioaccessibility were assessed. The release of peptide sequences during digestion was followed by LC–HRMS, and bioactive peptides were identified using a bioinformatic approach. At the end of digestion, fatty acid and protein bioaccessibility were similar in conventional and hyposodic PRC, but most of the bioactive peptides, mainly the ACE-inhibitors, were present in higher concentrations in the low-salt cheese. Considering that the sensory profiles were already evaluated as remarkably similar in conventional and hyposodic PRC, our results confirmed that shortening brine soaking time represents a promising strategy to reduce salt content in PRC.

## 1. Introduction

High salt intake is a key contributing factor for the prevalence of non-communicable diseases (NCDs) around the world. High-salt diets are linked to elevated blood pressure, a major risk factor for heart diseases and stroke, which in turn are among the leading causes of death worldwide [1,2]. Despite the current WHO recommendations for sodium (Na) consumption by adults (<2 g per day), in Europe the overall sodium intake varies between 2.7 and 7.1 g per day (7 to 18 g salt per day) in most countries [3], and processed foods might provide about 20% of the total Na intake [4]. Since salt reduction has been identified as one of the five priority interventions in response to the global NCD crisis [5], there is a growing interest in processing procedures lowering salt content, particularly when applied to highly appreciated and nutritional valued foods.

Parmigiano Reggiano cheese (PRC) is a long ripened, hard cheese made up of a combination of partially skimmed and whole raw milk, with the addition of a natural whey starter, principally consisting of thermophilic starting lactic acid bacteria [6]. The PRC is included in the list of foods with a Protected Designation of Origin (PDO), according to which the aging must last at least 12 months, starting from the forming of the cheese. The most valuable cheeses are those that ripen from 24 to 36 months, during which hydrolytic phenomena occur, and the organoleptic quality improves. EU regulation 1151/2012 ensures the method of production, quality, and area of origin of PDO products. In particular, PRC must be produced and transformed only in the Northern Italian provinces of Parma, Reggio Emilia, Modena, and in some parts of Bologna and Mantova. PRC is highly appreciated worldwide not only for its taste, but also for its nutritional characteristics [7]. PRC contains high-value nutritional proteins that are also functionally and biologically attractive, and their hydrolysates formed during ripening are supposed to be sources of bioactive peptides [8,9]. Recently, we investigated the impact of reducing the brining time in salt solution on PRC sensory properties and peptide content [10], but information is still lacking about possible modification of the bioaccessibility of protein and fatty acids, and on the release of biologically active peptides during digestion. Processing can deeply modify both nutrient content and bioaccessibility, i.e., the quantity of a compound that is released from the food matrix in the gastrointestinal tract, becoming available for intestinal absorption [11]. Modification in bioaccessibility due to changes in the supramolecular organization and in the network of interactions between molecules, or to nutrient localization within compartments may impact the nutritional value of food [12]. Indeed, in a previous work we reported the impact of different ripening time (15 and 30 months) on the kinetics of protein hydrolysis and the formation of peptides and small organic compounds during PRC in vitro digestion [13].

The aim of the present work was to evaluate whether the reduction of brine soaking time modifies fatty acid and protein bioaccessibility of a low-salt PRC produced and ripened for 30 months according to the PDO specification. To this aim, conventional (C-PRC) and hyposodic (Hypo-PRC) underwent in vitro static gastrointestinal digestion according to the INFOGEST protocol [14]. Sampling was performed at the end of the gastric phase, in the middle and at the end of the duodenal phase, and fatty acid and protein bioaccessibility was assessed. Digested samples were also analyzed by nuclear magnetic resonance (NMR) and liquid chromatography coupled with high resolution mass spectrometry (LC–HRMS), and the release of bioactive peptide sequences was verified using a bioinformatic approach.

## 2. Results

### 2.1. Fatty Acid Bioaccessibility

In agreement with Malacarne et al. [15], the main fatty acids found in undigested PRC were palmitic acid > oleic acid > myristic acid > stearic acid, which accounted for approximately 85% of total fatty acid with no differences between C- and Hypo-PRC (Supplementary Table S1). The release of fatty acids from the food matrix increased over time, and no differences were detected between C-PRC and Hypo-PRC at the end of digestion. However, the time course of fatty acid release was not the same in the two samples, bioaccessibility of myristic, stearic, oleic, total MUFA, and total fatty acids being higher in Hypo-PRC at D60 (Table 1).

### 2.2. Protein Bioaccessibility

**Figure 1:**
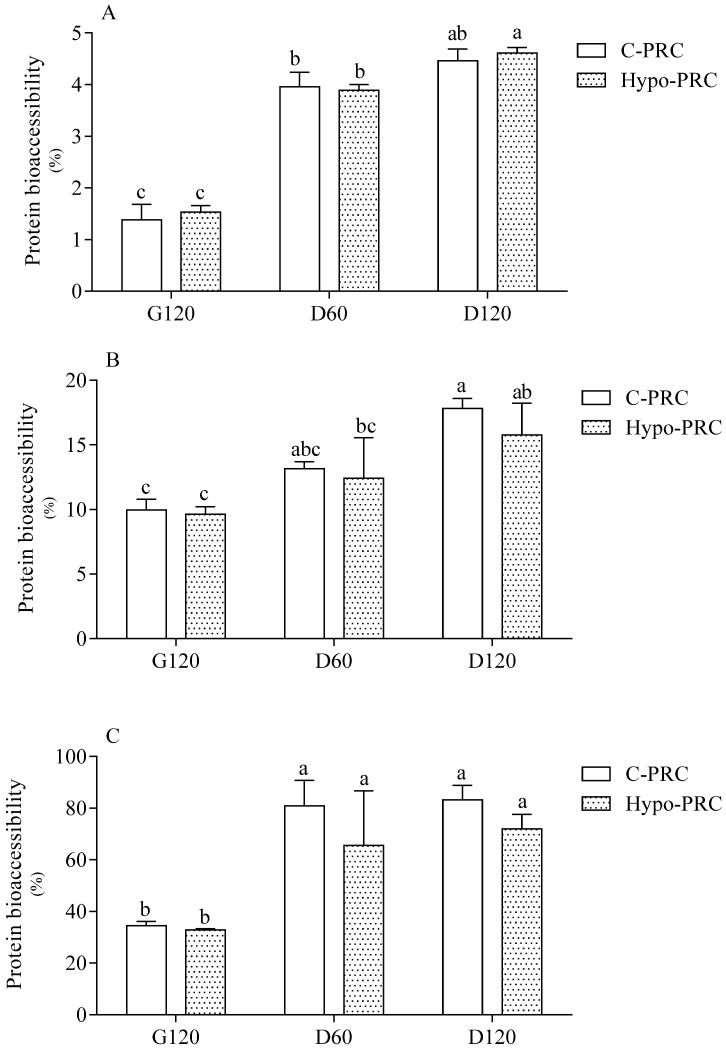
Protein bioaccessibility assessed by OPA (**A**) and Coomassie assay (**B**) and absorbance at 280 nm (**C**) in conventional Parmigiano Reggiano cheese (C-PRC) and hyposodic Parmigiano Reggiano cheese (Hypo-PRC) at different digestion times. Data are means ± SD of 3 independent in vitro digestions, each analyzed in triplicate. Protein bioaccessibility is expressed as % and it was calculated as the ratio × 100 between the protein mass in the digestion fluid and the total protein mass in the original undigested PRC. Statistical analysis was by the one-way ANOVA (A and C: *p* < 0.0001; B: *p* < 0.005) with Tukey’s post-hoc test (different letters indicate significant differences). G120: end of gastric phase; D60: 60 min of duodenal phase; D120: end of duodenal phase.

Protein bioaccessibility was evaluated by three different spectrophotometric methods (OPA, Coomassie, absorbance at 280 nm), all evidencing a time-dependent release of protein/peptides/amino acids from the matrix. Regardless of the analytical methodology, no differences were detected between C- and Hypo-PRC at any digestion time, and maximal bioaccessibility was already achieved at D60 (Figure 1).

### 2.3. HR-NMR Spectroscopy

**Figure 2:**
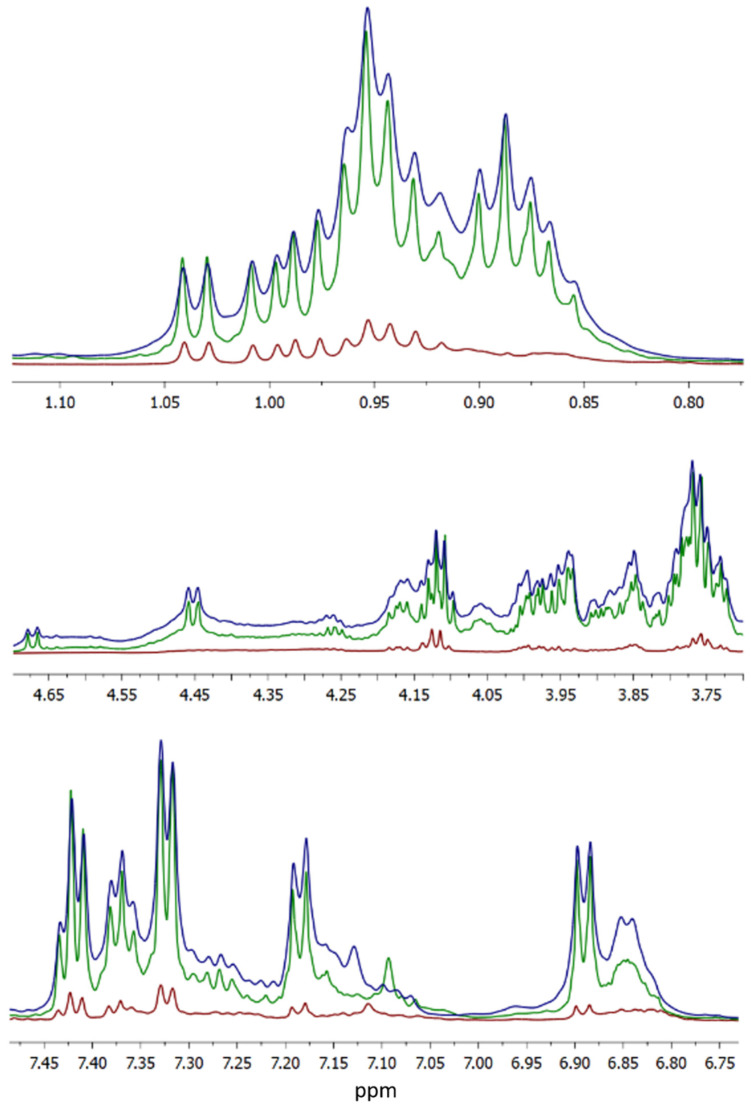
Proton HR-NMR spectrum of PRC digestion fluids at G120 (brown trace), D60 (green), and D120 (blue) phases. The (**top panel**) shows the upfield region, where mainly aliphatic hydrogen atoms of branched side chains of amino acids resonate. The (**middle panel**) shows the midfield spectral region, where hydrogen atoms bound to amino acids alpha-carbon resonate. Finally, the (**bottom panel**) shows the downfield spectral region, where hydrogen atoms belonging to aromatic amino acids resonate.

In Figure 2, the NMR spectrum of the digestion fluids after the three digestion phases is shown as traces with different colors, subdivided in three different spectral regions, collecting signals from hydrogen atoms located on aromatic, alpha-carbon, and aliphatic side chains of amino acids, respectively.

**Figure 3:**
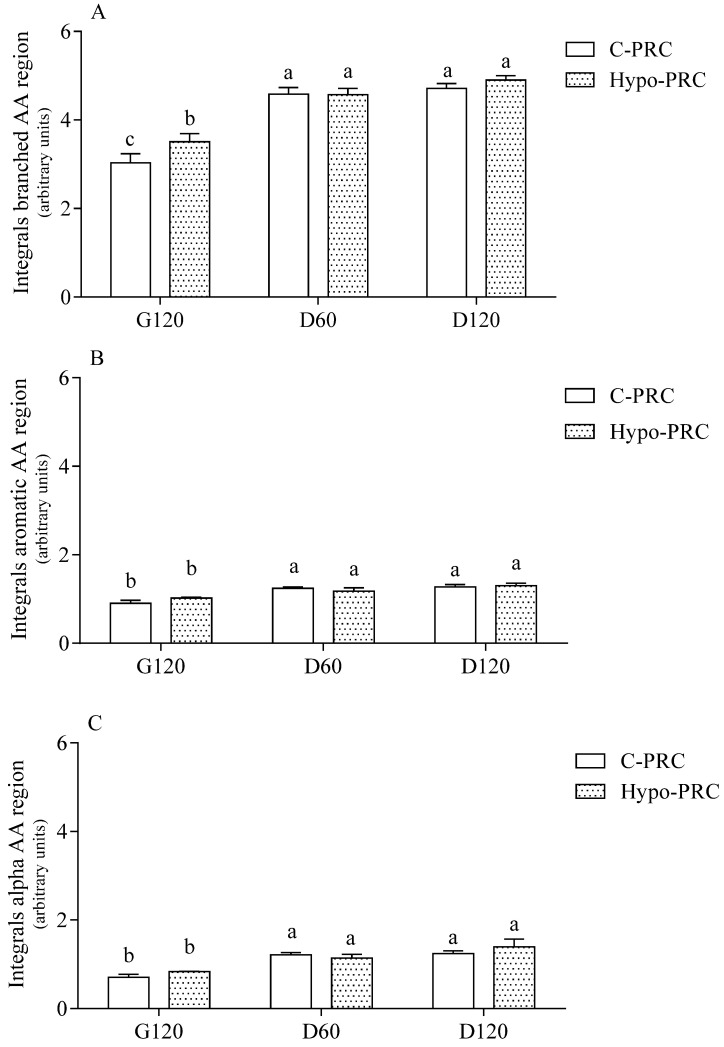
Integral area of branched (**A**), aromatic (**B**), and α-proton (**C**) amino acid region in conventional Parmigiano Reggiano cheese (C-PRC) and hyposodic Parmigiano Reggiano cheese (Hypo-PRC) at different digestion times. Data are means ± SD of 3 independent in vitro digestions, each one in triplicate. Integrals are expressed as arbitrary units. Statistical analysis was by the one-way ANOVA (all integrals *p* < 0.0001) with Tukey’s post-hoc test (different letters indicate significant differences). G120: end of gastric phase; D60: 60 min of duodenal phase; D120: end of duodenal phase.

More in detail, the progression of in vitro digestion of the two PRC evaluated by the appearance of NMR signal in branched, aromatic, and α-amino acid proton spectral regions is reported in Figure 3. The α-amino acid proton region includes the signals of all amino acids, both in the free state and bound to peptides or in soluble proteins. In each spectral region, the maximum release was already achieved at D60 with no significant difference between C- and Hypo-PRC except for branched amino acids, the release of which was higher in Hypo- than C-PRC at the end of the gastric digestion.

### 2.4. Peptide Formation

The total number of peptides generated at different digestion times from parent proteins was evaluated by LC–HRMS (Table 2). On average, fewer than 80 peptides were found in not-digested PRC, and in vitro digestion increased their number to >85 at G120 and >100 at D60 and D120. In both C- and Hypo-PRC, most of the peptides were from β-casein (60%) and αS1-casein (20%), whereas less than 10% of the identified sequences came from αS2-casein and κ-casein. The number of peptides from β-casein increased mainly during the duodenal phase, while those from κ-casein were mainly released during the gastric phase. Regarding whey proteins, a small number of peptides from β-lactoglobulin was released during the duodenal digestion. Overall, no significant differences were detected between C- and Hypo-PRC.

### 2.5. Bioinformatic Analysis

Bioactive sequences detected in not digested PRC and at different time points of in vitro digestion, their semiquantitative content, and their putative biological activity are reported in Table 3. Seven peptides with a documented bioactive sequence were found in not digested PRC, with no difference in their semiquantitative content between C- and Hypo-PRC. At the end of the gastric phase, bioactive peptides originally found in not digested samples were no longer detectable, or their concentration was significantly reduced, while new sequences were detected in both C- and Hypo-PRC, although in different amounts. All bioactive peptides detected at the end of the gastric phase were further hydrolyzed during the duodenal phase, and 15 peptides were detected in both C- and Hypo-PRC at the end of in vitro digestion. Among them, four (PQNIPPL, VYPFPGPI, LHLPLPL, PGPIPN) were more abundant in Hypo-PRC than C-PRC.

## 3. Discussion

Sodium chloride (NaCl) is one of the most widely used additives in food processing, and the adverse effects of high sodium (Na) consumption on blood pressure and NCD have been well documented [16,17]. The overall concern about Na dietary intake has boosted the development of new approaches to decrease the amount of salt added to food products [18,19]. In this study, we investigated the possible impact of an approximately 20% salt reduction (2.32 ± 0.06% dry matter (DM) and 1.90 ± 0.20% DM in C-PRC and Hypo-PRC, respectively) on the nutritional value of 30-months-ripened PRC. Indeed, the nutritional value of food does not exclusively depend on its chemical composition, as the bioaccessibility of nutrients is also a critical aspect to consider. The release/production of bioactive compounds, including bioactive peptides, upon digestion of protein-rich food is crucial as well. Since both bioaccessibility and release/generation of bioactive compounds can be affected by food processing [12,20,21], we evaluated fatty acids and protein bioaccessibility, as well as peptide formation in C-PCR and experimental Hypo-PCR.

The different salt concentrations modulated the kinetics of fatty acid release from the food matrix during in vitro digestion. This effect could reflect a higher hydrolysis degree of triacylglycerol during ripening. Salaberría et al. [22] already evidenced an accelerate lipolysis process in low-salt PRC. The faster lipolysis could be due to the decrease in the NaCl:moisture ratio, which could increase the amount of water available for triglyceride hydrolysis. However, at the end of in vitro digestion, fatty acid bioaccessibility was similar in C- and po-PRC.

Like fatty acids, protein bioaccessibility increased over time during digestion. Since our aim was to verify the time course and extent of protein hydrolysis during digestion, the use of the three spectrophotometric methods was mandatory since they selectively quantify proteins with different molecular weights. The evident disparity between the three absolute values of bioaccessibility reflected the powerlessness of the Coomassie assay to detect small peptides and amino acids [23], the latter instead detected by OPA assay [24]. Comparing results from the three methods, we assume that most proteins were hydrolyzed to fragments between 3000 Dalton and peptides bigger than five amino acids in length, which are detected by the absorbance in UV spectra only. The highest release of protein fragments below 3000 Dalton, a dimension compatible with peptide formation, was already reached in the middle of intestinal digestion (D60). The kinetics of protein hydrolysis and peptide/amino acid release was similar in C- and Hypo-PRC protein, which evidenced the same protein bioaccessibility at the end of the in vitro digestion.

Since the coexistence of soluble protein fragments larger than 3000 Dalton was still compatible with the above data, NMR spectroscopy was applied to verify their presence in the digested fractions. High resolution NMR spectroscopy provides information about the whole set of molecules present in solution if they have hydrogen atoms included in their structure, practically all organic molecules, including amino acids and their oligo-/polymers. A simple inspection of the resulting spectra provides quantitative and qualitative information such as (i) the solute concentrations, which are linearly correlated to the signal area independently from the molecule the hydrogen atom belongs to, (ii) the type of functional group hosting the identified atoms, for instance aromatic or aliphatic moieties, and (iii) the size or flexibility of the molecules which the atom is bound to. The latter property is revealed by the signal width: the broader the signal the larger and more rigid the molecule is, as expected by their shorter relaxation times, in turn reflected in the signal width [25]. The clear increment in the area of NMR signals demonstrated that digestion was hydrolyzing soluble fragments from insoluble proteins. It is worth noting that even in the last phase of digestion there were both narrow and broad signals, evidencing the co-presence of small peptides, with MW < 3 kDa and larger protein fragments with MW > 3 kDa. In not digested samples, the same bioactive peptides were present at similar concentration in both C- and Hypo-PRC. Among them, the sequences NLHLPLPLL (from β-CN, residues 132–140), LHLPLP (from β-CN, residues 133–138), and YKVPQL (from αS1-CN, residues 104–109) were already identified as ACE-inhibitory peptides in 12-months-ripened PRC [9], while the ACE-inhibitory and immunomodulating peptide LLYQEPVLGPVRGPFPIIV (β-CN 191–209) was detected in 24-months-ripened PRC extracts [26]. All peptides detected in not digested PRC were almost completely degraded during gastric digestion, and new bioactive sequences were released. In digested samples, most identified sequences derived from the hydrolysis of high molecular weight peptides already identified in undigested cheese [10] and from the cleavage of intact α- and β-casein and whey proteins. At the end of gastric digestion, a total of 10 bioactive peptides originated from β-casein, αS1-casein, and αS2-casein, confirming the high affinity of pepsin for these proteins [27,28]. Again, all peptides originated during gastric digestion were further hydrolyzed during the duodenal phase, which generated most of the bioactive peptides. Among these sequences, many fragments contained a PXP motif or a proline residue near to the carboxylic terminus. These structural motifs increase the resistance to the hydrolysis by digestive enzymes, which do not readily hydrolyze proline-containing peptides [29]. Most of the bioactive sequences detected at the end of the in vitro digestion were ACE-inhibitory. Among them, HLPLP (β-CN 134–138) and LHLPLPL (β-CN 133–139) originated from the hydrolysis of NLHLPLPLL (β-CN 132–140) and VENLHLPLPLL (β-CN 130–140) identified in not digested samples. Among bioactive peptides formed during duodenal digestion, and thus theoretically absorbed through the intestinal barrier, the sequences HLPLP, KEDVPSE, and LHLPLPL could elicit ACE-inhibitory activity.

Although the same bioactive peptides were detected in C- and Hypo-PRC, semiquantitative analysis revealed that most of them were present at higher concentration in Hypo-PRC at the end of digestion. A previous in vitro study performed on myoglobin evidenced that NaCl reduced protein digestibility, promoting the exposure of hydrophobic amino acids, making the binding of phenylalanine with its surrounding environment within the protein core stronger, and eventually resulting in a protein organization less prone to undergoing enzyme-dependent hydrolysis [30]. However, it is not easy to explain how salt concentration selectively modulated the formation of biologically active peptides during gastrointestinal digestion, and further studies are needed to clarify this important aspect. To the best of our knowledge, the present study is the first one investigating the kinetics of bioactive fragment formation during the different phases of digestion. Indeed, although the evolution of bioactive peptide formation with ripening was already addressed [26], and several studies reported bioactive peptides released from PRC after in vitro digestion [26,31,32], exhaustive information regarding the fate of these peptides was still lacking. Our results represent a further step to uncover the hidden functionality of foods that is linked to bioactive peptide formation.

Based on the herein reported results, we can conclude that reduction of salt content in ripened PRC did not significantly affect fatty acid and protein bioaccessibility and led to the formation of a higher number of bioactive peptides after gastrointestinal digestion. Of note, ACE-inhibitors peptides were more abundant in Hypo- than C-PRC. The presence of these peptides has been reported in various fermented milk products, the antihypertensive effect of which has been proved by various in vitro and in vivo (animal and human trials) experiments [33]. Considering that in a previous study we evidenced that the sensory profiles were remarkably similar in C- and Hypo-PRC, without any off-flavor development [10], our results confirmed that reduction of salt content in ripened PRC represents a promising strategy to reduce Na intake, potentially protecting consumers’ health. In this light, the evaluation of the actual ACE-inhibitory activity of digested C- and Hypo-PRC deserves further studies in biological systems.

## 4. Materials and Methods

### 4.1. Materials

Unless specified, chemicals and solvents were of the highest analytical grade and purchased from Merck (Darmstadt, Germany) and Sigma-Aldrich (St. Louis, MO, USA).

### 4.2. Parmigiano Reggiano Cheese (PRC)

PRC was produced and ripened according to the Protection Designation of Origin specification, which includes restrictions to its geographical area of production, cow feeding, and cheese manufacturing. PRC was produced in a local dairy by using conventional (18 days, 3 samples from 3 different wheels) and reduced (12 days, 3 samples from 3 different wheels) brine soaking times in a saturated sodium chloride solution (about 36% *w/w*). Average salt content was 2.32 ± 0.06% dry matter (DM) and 1.90 ± 0.20% DM in C-PRC and short brine soaking time Hypo-PRC, respectively (*p* = 0.0253). A detailed description of PRC production and proximate analysis of C-PRC and Hypo-PRC is reported in a previous work [10].

### 4.3. In Vitro Digestion

In vitro digestion was performed in triplicate according to the INFOGEST protocol [14]. To simulate chewing, PRC was chopped before starting oral digestion. In vitro digestion lasted for 242 min (2 min of oral digestion, 120 min of gastric digestion, and 120 min of intestinal digestion) at 37 °C. During the process, several consecutive enzymatic reactions took place by the addition of simulated saliva, simulated gastric juice (containing 2000 U/mL pepsin) at pH 3, and simulated pancreatic juice (containing 10 mM bile and 100 U/mL pancreatin) at pH 7. Samples were taken at the end of the gastric phase (G120), after 60 min (D60), and at the end of the duodenal phase (D120). Digested samples were centrifuged at 50,000× *g* for 15 min. To remove any turbidity, supernatants were filtered with 0.2 μm membranes and stored at −80 °C until further analysis.

### 4.4. Fatty Acid Bioaccessibility

Total lipids were extracted according to Bligh and Dyer [34], with slight modifications. Briefly, 6 mL of methanol, 3 mL of chloroform, and 2.4 mL of distilled water were sequentially added to 0.1 g of food or 0.8 mL of digested sample followed by thorough mixing with magnetic stirring at the maximum intensity for 3 min. Successively, 3 mL of chloroform and 3 mL of distilled water, each one followed by thorough mixing with magnetic stirring for 1 min, were added. The solution was kept overnight at 4 °C, and then the upper layer was removed by suction, and the lower chloroform layer was transferred to a test tube. Pentadecanoic acid (1 mg) was added as internal standard, and the chloroform layer was dried under nitrogen infusion. After methylation [35], the quantitative and qualitative content of fatty acids (as methyl esters—FAMEs) was determined by fast-GC (GC-2030AF; Shimadzu, Kyoto, Japan) using a capillary column (30 mt, 0.2 μm film thickness) with a programmed temperature gradient (50–250 °C, 10 °C/min). The gas chromatographic peaks were identified based on their retention time ratios relative to methyl stearate and predetermined by use of authentic samples [36]. Gas chromatographic traces and quantitative evaluations were obtained using Lab Solution software (Shimadzu, Kyoto, Japan) and normalized for the dilution factor due to the addition of digestive fluids. FAMEs from chemicals added during in vitro digestion system were subtracted, and bioaccessibility was calculated as FAME concentration in digested sample/FAME concentration in PRC before digestion × 100 [37].

### 4.5. Protein Bioaccessibility

Protein concentration was determined spectrophotometrically by o-phthaldialdehyde (OPA) [38] and Coomassie assay [39], and by measuring the absorbance at 280 nm [40] using L-isoleucine, bovine serum albumin, and non-fat dry milk as standards, respectively. Values were normalized for the dilution factor due to the addition of digestive fluids, and protein content from enzymes added during in vitro digestion was subtracted. Bioaccessibility was calculated as protein mass in digested sample/protein mass in PRC before digestion × 100 [37].

### 4.6. HR-NMR Spectroscopy

HR-NMR analysis was performed in digested sample as previously reported [41], with slight modification. Samples were thawed, centrifuged first at 2300× *g* for 5 min at 4 °C to eliminate the coarser particles, and then at 50,000× *g* for 5 min at 4 °C to eliminate the fine particulates. Afterward, each sample was vortexed for 30 s, then 750 μL of supernatant was taken and added to 120 μL of 100 mM phosphate buffer with 10 mM trimethylsilylpropanoic acid (TSP), molecular weight (MW) 172.27 g/mol (Cambridge Isotope Lab Inc., Tewksbury, MA, USA) and brought to pH 7.0. HR-NMR spectra were recorded at 298 K on a Bruker US+ Avance III spectrometer operating a proton frequency of 600.13 MHz, equipped with a BBI-z probe and a SampleCase™ sampler for automation. The spectra were collected with a 90° pulse of 13.1 μs with 10 W of power, a relaxation delay of 5 s, and an acquisition time of 2.3 s; for each sample 256 scans were obtained, resulting in 32 K data points over 7,194,245 Hz encompassing a spectral width of 12 ppm. The residual signal of monodeuterated water (HOD) was suppressed using the NOESYGPPR1D sequence (a standard pulse sequence provided in the Bruker library), which incorporated the first increment of the NOESY pulse sequence plus a spoil gradient. A Fourier transform was exploited to extract the frequency–domain spectrum of 64 K data points from the raw time–domain FID. TopSpin version 3.5.6 was used to automatically execute phase and base line fixes (Bruker BioSpin, Karlsruhe, Germany). The chemical shifts were internally referenced to the DSS at 0.000 ppm. Data are expressed as arbitrary units [12].

### 4.7. Bioactive Peptide Release and Identification

Digested samples were centrifuged at 14,000 rpm for 40 min at 4 °C with a 5810 R centrifuge (Eppendorf s.r.l, Milan, Italy) to remove any particulate matter formed during freezing/thawing. The supernatants were filtered through 0.45 µm PTFE filters and directly injected in the UPLC–MS system. Samples were separated by a reverse-phase Acquity UPLC Peptide BEH 300 C18 column (1.7 µm, 2.1 × 150 mm, Waters, Milford, MA, USA) in a UPLC system coupled with ESI and MS (UPLC Acquity I-class, with a Vion IMS QTof Mass Spectrometer, Waters, Milford, MA, USA). Gradient elution was set as follows with eluent A (H_2_O with 0.2% CH_3_CN and 0.1% HCOOH) and eluent B (CH_3_CN with 0.1% HCOOH)): 0 to 7 min, isocratic 100% A; 7 to 50 min, linear gradient from 100% A to 50% A; 50–52.6 min 50% A, 52.6–53 min from 50% A to 0% A, 53–58.2 min 0% A, 58.2–59 min from 0% A to 100% A, 59–72 min 100% A and reconditioning. Flow rate was set at 0.20 mL min^–1^, injection volume 1 μL, column temperature 35 °C, sampler temperature 18 °C, and the total run time was 72 min. Detection was performed using a Vion IMS QTof mass spectrometer (Waters, Milford, MA, USA) with the following parameters. Experiment type: peptide map (IMS), experiment type: MSe, source type: ESI, polarity: positive, analyzer mode: sensitivity, mode: standard transmission, capillary: 3.00 kV, sample cone voltage: 40 V, source offset voltage: 80 V, source temperature: 120 °C, desolvation temperature: 450 °C, cone gas flow: 50 L/h, desolvation gas flow: 800 L/h. MSe mode: high definition MSe, acquisition time: 0–58.2 min, scan range: 100–2000 *m*/*z*, scan time: 0.4 s, low collision energy: 6 V, high collision energy ramp: 20 to 45 V, automatic lock correction (leucine enkephaline). The software used for data processing was UNIFI (Waters Corporation, Milford MA, USA). The expected component list comprised the following protein Uniprot accession numbers: P02666 (β-casein), P02662 (αS1-casein), P02663 (αS2-casein), P02668 (κ-casein), P02754 (β-lactoglobulin), P00711 (α-lactalbumin). Variable amino acid modifications were included as deamidation (N, Q), pyroglutamic acid N-term (E, Q), oxidation (single or double, M or W), phosphorylation (S, T, Y). Peptide semiquantitative data were obtained normalizing integral areas.

Released bioactive peptides were identified using a bioinformatic approach. The whole set of peptide sequences under analysis was searched into two benchmark databases of peptide bioactivity, namely BIOPEP (http://www.uwm.edu.pl/biochemia/index.php/en/biopep, (accessed on 9 July 2021)) [42] and AHTPDB (http://crdd.osdd.net/raghava/ahtpdb/, (accessed on 9 July 2021)) [43], which were queried automatically and systematically employing a script pipeline developed “in-house”.

### 4.8. Statistical Analysis

Statistical differences were determined by the one-way analysis of variance (ANOVA) followed by Tukey’s post hoc test using Prism software ver. 7.0 (GraphPad, San Diego, CA, USA). Different letters indicate significant differences (at least *p* < 0.05).

## Figures and Tables

**Table 1:** Fatty acid bioaccessibility in conventional Parmigiano Reggiano cheese (C-PRC) and hyposodic Parmigiano Reggiano cheese (Hypo-PRC) at different digestion times.

	G120		D60		D120	
	C-PRC	Hypo-PRC	C-PRC	Hypo-PRC	C-PRC	Hypo-PRC
C8:0	1.53 ± 0.58 b	1.87 ± 0.12 b	11.70 ± 3.73 ab	1.08 ± 1.53 b	16.32 ± 6.09 a	16.69 ± 1.82 a
C10:0	1.24 ± 0.34 c	2.05 ± 0.16 c	22.18 ± 3.55 ab	16.76 ± 6.50 b	31.36 ± 4.48 a	32.10 ± 3.63 a
C12:0	1.55 ± 0.17 c	2.51 ± 0.11 c	27.32 ± 4.78 b	35.60 ± 3.62 ab	42.51 ± 4.30 a	38.21 ± 1.20 a
C14:0	1.48 ± 0.23 c	3.00 ± 0.05 c	26.30 ± 4.92 b	45.17 ± 1.24 a	43.99 ± 9.63 ab	34.19 ± 5.46 ab
C16:0	1.52 ± 0.38 b	2.89 ± 0.02 b	22.32 ± 4.81 ab	43.83 ± 5.05 a	39.80 ± 11.00 a	28.24 ± 4.79 a
C16:1 n-7	0.00 ± 0.00 c	1.32 ± 1.86 c	33.92 ± 5.47 b	49.02 ± 3.33 ab	53.47 ± 9.05 a	44.45 ± 4.37 ab
C18:0	1.84 ± 0.37 c	2.88 ± 0.08 c	20.68 ± 5.26 bc	43.23 ± 5.89 a	37.24 ± 10.12 a	25.30 ± 5.6 ab
C18:1 n-9	0.96 ± 0.74 c	2.03 ± 0.15 c	27.99 ± 6.26 b	45.29 ± 6.01 a	47.79 ± 5.10 a	47.86 ± 3.24 a
C18:2 n-6	0.53 ± 0.92 c	1.07 ± 1.52 c	34.01 ± 5.20 b	47.58 ± 7.64 ab	56.43 ± 9.48 a	48.29 ± 4.37 ab
ΣSFA	1.47 ± 0.30 b	2.69 ± 0.05 b	21.72 ± 4.69 a	39.12 ± 2.00 a	37.58 ± 8.64 a	27.95 ± 4.27 a
ΣMUFA	0.88 ± 0.68 c	1.98 ± 0.00 c	28.40 ± 6.24 b	45.60 ± 5.76 a	48.22 ± 5.38 a	47.60 ± 2.69 a
ΣPUFA	0.53 ± 0.92 c	1.07 ± 1.52 c	34.01 ± 5.20 b	47.58 ± 7.64 ab	56.43 ± 9.48 a	48.29 ± 4.37 ab
Total	1.28 ± 0.40 c	2.45 ± 0.07 c	23.95 ± 5.13 b	41.13 ± 3.15 a	40.97 ± 7.59 a	33.90 ± 2.48 ab

Data are means ± SD of three independent in vitro digestions, each one in duplicate. Fatty acid bioaccessibility is expressed as % and was calculated as fatty acid methyl ester concentration in digested/fatty acid methyl ester concentration in PRC before digestion × 100. Statistical analysis was by one-way ANOVA (C8:0: *p* = 0.0017; C16:0: *p* = 0.0002; C10:0, C12:0, C14:0, C16:1 n-7, C18:0, C18:1 n-9, C18:2 n-6, ΣSFA, ΣMUFA, ΣPUFA and total fatty acids: *p* < 0.0001) with Tukey’s post-hoc test (different letters indicate significant differences). G120: end of gastric phase; D60: 60 min of duodenal phase; D120: end of duodenal phase.

**Table 2:** Number of different peptides released in conventional Parmigiano Reggiano cheese (C-PRC) and hyposodic Parmigiano Reggiano cheese (Hypo-PRC) at different digestion times.

Protein Source	Not digested ^a^		G120		D60		D120		ANOVA
	C-PRC	Hypo-PRC	C-PRC	Hypo-PRC	C-PRC	Hypo-PRC	C-PRC	Hypo-PRC	
β-casein	41.0 ± 0.0 c	41.0 ± 0.0 c	46.33 ± 2.52 c	50.0 ± 2.0 bc	66.0 ± 5.2 a	60.0 ± 3.46 ab	65.33 ± 9.45 a	66.0 ± 2.0 a	*p* < 0.0001
αS1-casein	25.0 ± 0.0 a	25.0 ± 0.0 a	19.67 ± 0.58 a	21.67 ± 1.15 a	25.0 ± 3.61 a	23.3 ± 2.61 a	21.67 ± 3.79 a	24.33 ± 3.06 a	*p* = 0.0992
αS2-casein	9.0 ± 0.0 a	9.0 ± 0.0 a	7.0 ± 1.0 ab	7.33 ± 1.15 ab	6.67 ± 0.58 b	8.33 ± 0.58 ab	7.33 ± 1.53 ab	8.0 ± 0.0 ab	*p* = 0.0167
κ-casein	3.0 ± 0.0 c	3.0 ± 0.0 c	9.33 ± 0.58 a	9.67 ± 1.15 a	3.33 ± 0.58 c	6.0 ± 1.0 b	3.33 ± 1.15 c	3.67 ± 0.58 c	*p* < 0.0001
β-LG(A and B isoforms)	0.0 ± 0.0 b	0.0 ± 0.0 b	0.0 ± 0.0 b	0.0 ± 0.0 b	1.0 ± 0.0 a	0.67 ± 0.58 ab	1.0 ± 0.0 a	0.67 ± 0.58 ab	*p* = 0.0004
Total	78.0 ± 0.0 c	78.0 ± 0.0 c	82.33 ± 2.31 bc	88.67 ± 4.16 ABC	102.0 ± 8.72 a	98.0 ± 6.56 ab	98.67 ± 12.50 ab	102.67 ± 4.93 a	*p* = 0.0002

^a^ [10]. Data are means ± SD of three different PRC and independent in vitro digestions. Statistical analysis was performed by one-way ANOVA with Tukey’s post-hoc test (different letters indicate significant differences). G120: end of gastric phase; D60: 60 min of duodenal phase; D120: end of duodenal phase; LG: lactoglobulin.

**Table 3:** Relative bioactive peptide abundance in conventional Parmigiano Reggiano cheese (C-PRC) and hyposodic Parmigiano Reggiano cheese (Hypo-PRC) at different digestion times.

Peptide Sequence	Protein Source	Not Digested		G120		D60		D120		Reported Activity (uM IC_50_)
		C-PRC	Hypo-PRC	C-PRC	Hypo-PRC	C-PRC	Hypo-PRC	C-PRC	Hypo-PRC	
LHLPLP	β-CN (133–138)	62 ± 3.8 a	64.7 ± 7.8 a	0.0 ± 0.0 b	0.0 ± 0.0 b	0.0 ± 0.0 b	0.0 ± 0.0 b	0.0 ± 0.0 b	0.0 ± 0.0 b	ACE inhibition (5.5)
RPKH	αS1-CN (1–4)	0.9 ± 0.1 a	0.9 ± 0.1 a	0.0 ± 0.0 b	0.0 ± 0.0 b	0.0 ± 0.0 b	0.0 ± 0.0 b	0.0 ± 0.0 b	0.0 ± 0.0 b	ACE inhibition (>1863)
YKVPQL	αS1-CN (104–109)	0.6 ± 0.1 a	0.6 ± 0.1 a	0.0 ± 0.0 b	0.0 ± 0.0 b	0.0 ± 0.0 b	0.0 ± 0.0 b	0.0 ± 0.0 b	0.0 ± 0.0 b	ACE inhibition (>22)
NLHLPLPLL	β-CN (132–140)	25.2 ± 1.9 a	25.5 ± 2.7 a	0.0 ± 0.0 b	0.0 ± 0.0 b	0.0 ± 0.0 b	0.0 ± 0.0 b	0.0 ± 0.0 b	0.0 ± 0.0 b	ACE inhibition (15)
RELEEL	β-CN (1–6)	1.5 ± 0.4 ab	2.0 ± 0.5 a	0.9 ± 0.1 b	0.0 ± 0.0 c	0.0 ± 0.0 c	0.0 ± 0.0 c	0.0 ± 0.0 c	0.0 ± 0.0 c	ACE inhibition (1000)
LLYQEPVLGPVRGPFPIIV	β-CN (191–209)	2.6 ± 0.3 a	2.7 ± 0.3 a	0.3 ± 0.0 b	0.3 ± 0.0 b	0.0 ± 0.0 b	0.0 ± 0.0 b	0.0 ± 0.0 b	0.0 ± 0.0 b	ACE inhibition andimmunomodulating (nr)
RPKHPIKHQGLPQEVLNENLLRF	αS1-CN (1–23)	4.7 ± 1.3 a	5.22 ± 1.2 a	0.3 ± 0.1 b	0.3 ± 0.1 b	0.0 ± 0.0 b	0.0 ± 0.0 b	0.0 ± 0.0 b	0.0 ± 0.0 b	Antibacterial (nr)
AYFYPEL	αS1-CN (143–149)	0.0 ± 0.0 c	0.0 ± 0.0 c	0.26 ± 0.0 a	0.22 ± 0.04 b	0.0 ± 0.0 c	0.0 ± 0.0 c	0.0 ± 0.0 c	0.0 ± 0.0 c	ACE inhibition (6.6)
DAYPSGAW	αS1-CN (157–164)	0.0 ± 0.0 b	0.0 ± 0.0 b	0.0 ± 0.0 b	0.05 ± 0.01 a	0.0 ± 0.0 b	0.0 ± 0.0 b	0.0 ± 0.0 b	0.0 ± 0.0 b	ACE inhibition (98)
LKKISQRYQKFALPQYLKT	αS2-CN (164–182)	0.0 ± 0.0 c	0.0 ± 0.0 c	1.5 ± 0.0 a	1.4 ± 0.06 b	0.0 ± 0.0 c	0.0 ± 0.0 c	0.0 ± 0.0 c	0.0 ± 0.0 c	Hemolytic (nr)
PLW	αS1-CN (196–199)	0.0 ± 0.0 b	0.0 ± 0.0 b	0.1 ± 0.01 a	0.0 ± 0.0 b	0.0 ± 0.0 b	0.0 ± 0.0 b	0.0 ± 0.0 b	0.0 ± 0.0 b	ACE inhibition (36)
VENLHLPLPLL	β-CN (129–139)	0.0 ± 0.0 c	0.0 ± 0.0 c	0.4 ± 0.01 a	0.35 ± 0.03 b	0.0 ± 0.0 c	0.0 ± 0.0 c	0.0 ± 0.0 c	0.0 ± 0.0 c	Anticancer (nr)
VYQHQKAMKPWIQPKTKVIPYVRYL	αS2-CN (183–207)	0.0 ± 0.0 b	0.0 ± 0.0 b	2.3 ± 0.4 a	2.4 ± 0.1 a	0.0 ± 0.0 b	0.0 ± 0.0 b	0.0 ± 0.0 b	0.0 ± 0.0 b	Antibacterial (nr)
YQKFPQY	αS2-CN (89–95)	0.0 ± 0.0 a	0.0 ± 0.0 a	0.8 ± 0.08 a	0.7 ± 0.0 b	0.0 ± 0.0 c	0.0 ± 0.0 c	0.0 ± 0.0 c	0.0 ± 0.0 c	ACE inhibition (20.1)
TPEVDDEALEK	β-Lg (125–135)	0.0 ± 0.0 c	0.0 ± 0.0 c	0.0 ± 0.0 c	0.0 ± 0.0 c	0.4 ± 0.05 a	0.2 ± 0.0 b	0.0 ± 0.0 c	0.0 ± 0.0 c	DPP-IV inhibition (319.5)
VPYPQ	β-CN (177–181)	0.0 ± 0.0 b	0.0 ± 0.0 b	0.0 ± 0.0 b	0.0 ± 0.0 b	0.1 ± 0.01 a	0.1 ± 0.01 a	0.0 ± 0.0 b	0.0 ± 0.0 b	Antioxidant (nr)
FYPEL	αS1-CN (145–149)	0.0 ± 0.0 b	0.0 ± 0.0 b	0.0 ± 0.0 b	0.0 ± 0.0 b	0.0 ± 0.0 b	0.7 ± 0.0 a	0.0 ± 0.0 b	0.0 ± 0.0 b	Antioxidant (nr)
HLPLP	β-CN (134–138)	0.0 ± 0.0 b	0.0 ± 0.0 b	0.0 ± 0.0 b	0.0 ± 0.0 b	0.5 ± 0.2 b	1.7 ± 0.8 a	0.4 ± 0.08 b	0.4 ± 0.1 b	ACE inhibition (41)
EMPFPK	β-CN (108–113)	0.0 ± 0.0 b	0.0 ± 0.0 b	0.0 ± 0.0 b	0.0 ± 0.0 b	2.5 ± 0.4 a	2.8 ± 0.4 a	2.7 ± 0.1 a	2.4 ± 0.1 a	ACE inhibition (423)
HLPLPL	β-CN (134–139)	0.0 ± 0.0 b	0.0 ± 0.0 b	0.0 ± 0.0 b	0.0 ± 0.0 b	0.5 ± 0.2 b	1.7 ± 0.8 a	0.4 ± 0.08 b	0.4 ± 0.1 b	Antiamnestic (10)
KEDVPSE	αS1-CN (83–89)	0.0 ± 0.0 c	0.0 ± 0.0 c	0.0 ± 0.0 c	0.0 ± 0.0 c	0.06 ± 0.0 b	0.1 ± 0.04 a	0.1 ± 0.0 c	0.1 ± 0.0 c	ACE inhibition (41)
LPLPL	β-CN (135–139)	0.0 ± 0.0 c	0.0 ± 0.0 c	0.0 ± 0.0 c	0.0 ± 0.0 c	0.8 ± 0.3 b	1.8 ± 0.3 a	1.1 ± 0.2 b	1.1 ± 0.3 b	DPP-IV inhibition (325)
LPYP	k-CN (56–59)	0.0 ± 0.0 c	0.0 ± 0.0 c	0.0 ± 0.0 c	0.0 ± 0.0 c	1.1 ± 0.3 ab	1.5 ± 0.1 a	1.1 ± 0.2 ab	0.9 ± 0.2 b	ACE inhibition (5)
NIPPLTQTPV	β-CN (73–82)	0.0 ± 0.0 c	0.0 ± 0.0 c	0.0 ± 0.0 c	0.0 ± 0.0 c	0.9 ± 0.2 ab	0.5 ± 0.09 bc	1.3 ± 0.6 a	0.9 ± 0.2 ab	ACE inhibition (173)
PQNIPPL	β-CN (71–77)	0.0 ± 0.0 d	0.0 ± 0.0 d	0.0 ± 0.0 d	0.0 ± 0.0 d	0.02 ± 0.0 c	0.05 ± 0.01 b	0.025 ± 0.0 c	0.1 ± 0.01 a	DPP-IV inhibition (1500)
VVPPFLQPE	β-CN (83–91)	0.0 ± 0.0 b	0.0 ± 0.0 b	0.0 ± 0.0 b	0.0 ± 0.0 b	1.5 ± 0.1 a	1.6 ± 0.6 a	1.5 ± 0.5 a	2.0 ± 0.2 a	Antioxidant (nr)
VYPFPGPI	β-CN (59–66)	0.0±0.0 d	0.0 ± 0.0 d	0.0 ± 0.0 d	0.0 ± 0.0 d	2.9 ± 0.2 c	4.0 ± 0.2 b	4.2 ± 0.6 b	5.2 ± 0.4 a	Antiamnestic (110)
YPEL	αS1–CN (146-149)	0.0 ± 0.0 b	0.0 ± 0.0 b	0.0 ± 0.0 b	0.0 ± 0.0 b	1.4 ± 0.2 a	1.5 ± 0.2 a	1.2 ± 0.1 a	1.3 ± 0.1 a	Antioxidant (nr)
YPFPGPI	β-CN (60–66)	0.0 ± 0.0 b	0.0 ± 0.0 b	0.0 ± 0.0 b	0.0 ± 0.0 b	0.8 ± 0.06 a	1.1 ± 0.4 a	0.9 ± 0.1 a	0.8 ± 0.007 a	ACE inhibition (500)
YPVEPF	β-CN (114–119)	0.0 ± 0.0 b	0.0 ± 0.0 b	0.0 ± 0.0 b	0.0 ± 0.0 b	7.6 ± 1.0 a	9.0 ± 1.8 a	7.7 ± 0.8 a	8.7 ± 0.8 a	Opioid (nr)
LHLPLPL	β-CN (133–139)	0.0 ± 0.0 c	0.0 ± 0.0 c	0.0 ± 0.0 c	0.0 ± 0.0 c	0.0 ± 0.0 c	0.0 ± 0.0 c	0.2 ± 0.09 b	0.3 ± 0.03 a	ACE inhibition (432.7)
PGPIPN	β-CN (63–68)	0.0 ± 0.0 b	0.0 ± 0.0 b	0.0 ± 0.0 b	0.0 ± 0.0 b	0.0 ± 0.0 b	0.0 ± 0.0 b	0.0 ± 0.0 b	0.09 ± 0.0 a	Immunomodulating (nr)

Data are means ± SD of three samples. Semiquantitative content was performed normalizing single peptide area against the sum of all peptide areas. Statistical analysis was performed by one-way ANOVA (all peptides *p* < 0.0001) with Tukey’s post-hoc test (different letters indicate significant differences). ACE: angiotensin converting enzyme; DPP-IV: dipeptidyl peptidase 4; G120: end of gastric phase; D60: 60 min of duodenal phase; D120: end of duodenal phase.

## Data Availability

No applicable.

## Supplementary Materials

The following supporting information can be downloaded. Table S1: Fatty acid methyl ester content in conventional Parmigiano Reggiano cheese (C-PRC) and hyposodic Parmigiano Reggiano cheese (Hypo-PRC).

## Sample Availability

Samples of the compounds are not available from the authors.
