# Supplementary material for: Impact of a Shorter Brine Soaking Time on Nutrient Bioaccessibility and Peptide Formation in 30-Months-Ripened Parmigiano Reggiano Cheese
Source: Molecules. 2022 Jan 20;27(3):664. doi: 10.3390/molecules27030664 (PMC8840655; doi:10.3390/molecules27030664)
Supplement: Supplementary file 1 [file molecules-27-00664-s001.zip › molecules-1519883-supplementary.pdf]

Supplementary Table S1. Fatty acid methyl esters content in conventional Parmigiano-Reggiano cheese (C-PRC) and hyposodic Parmigiano-Reggiano cheese (Hypo-PRC).

	**C-PRC**	**Hypo-PRC**
C8:0	216.37±16.58	220.90±43.74
C10:0	552.78±28.06	563.87±81.72
C12:0	684.14±29.52	671.87±70.75
C14:0	2191.56±151.62	2120.22±115.49
C16:0	5993.19±350.51	5832.99±330.59
C16:1 n-7	350.49±14.09	349.37±49.00
C18:0	1807.86±497.96	1789.42±295.13
C18:1 n-9	4286.09±634.15	4288.07±104.86
C18:2 n-6	505.73±124.86	505.97±64.26
ΣSFA	11445.91±968.33	11199.26±245.87
ΣMUFA	4636.58±624.78	4637.44±98.28
ΣPUFA	505.73±124.86	505.97±64.26
Total	16588.22±1709.90	16342.67±369.40

Data are means ± SD of three independent analysis and are expressed as mg of fatty acid methyl esters/100g PRC. Statistical analysis was by evaluated by unpaired t-test (* at least $p<0.05$).
